# Perioperative Avulsion of a Left Internal Mammary Artery Graft in a Patient with Syphilis

**DOI:** 10.1155/2014/574346

**Published:** 2014-01-02

**Authors:** Vasily I. Kaleda, Sergei A. Belash, Alexei V. Barsuk, Kirill O. Barbuhatti

**Affiliations:** ^1^Department of Adult Cardiac Surgery, Ochapowski Regional Hospital #1, Chest Surgery Center, 140, Rossiyskaya Street, Krasnodar 350086, Russia; ^2^Pathomorphology Lab, Ochapowski Regional Hospital #1, Chest Surgery Center, 140, Rossiyskaya Street, Krasnodar 350086, Russia

## Abstract

Avulsion of a graft after coronary artery bypass grafting surgery is a rare but very serious complication which leads to massive bleeding and possible life-threatening cardiac tamponade. In this paper we report a very rare case of a left internal mammary artery graft avulsion on the day of surgery in a patient with syphilis.

## 1. Introduction

Avulsion of a graft after coronary artery bypass grafting (CABG) surgery is a rare but very serious complication which leads to massive bleeding and possible life-threatening cardiac tamponade. It was reported to occur after minimally invasive direct coronary artery bypass (MIDCAB) surgery due to a graft tension and after conventional CABG due to mediastinitis. In this paper we report a very rare case of a left internal mammary artery (LIMA) graft avulsion on the day of CABG surgery in a patient with syphilis.

## 2. Case Report

A 54-year-old male patient presented with 3rd CCS class angina pectoris. Risk factors of coronary artery disease included class I obesity, hypertension, hyperlipidemia, and smoking. Transthoracic echocardiography showed normal size of the ascending aorta with a slight thickening of its wall, normal aortic valve function without regurgitation, and slightly decreasedleft ventricular ejection fraction with local hypokinesia. Pulmonary examination revealed no evidence of chronic obstructive pulmonary disease or marked emphysema. Coronary angiography showed multivessel lesions including proximal left anterior descending artery (LAD) stenosis and occlusion of left circumflex and posterior descending (PDA) arteries. There was no evidence of any arteritis or connective tissue disorder. The patient denied any history of sexually transmitted infection, but preoperative screening for syphilis with rapid plasma reagin (RPR) test was positive (this test is a part of standard examination before surgery in Russia). It was then confirmed with enzyme immunoassay (EIA) test, *Treponema pallidum* passive particle agglutination assay (TPPA) test, and fluorescent treponemal antibody absorption (FTA-ABS) test. The skin and visible mucous membranes were free from any sign of syphilis. The patient was suspected to have latent syphilis and was offered surgery.

CABG was performed by experienced staff surgeon (S.A.B.) using cardiopulmonary bypass (CPB) and cold crystalloid cardioplegia with LIMA to LAD and vein grafts to obtuse marginal artery and PDA. LIMA was dissected from its origin to the bifurcation using pedicled in situ technique, and after cutting off it had a satisfactory blood flow. There was no doubt in the good quality of the graft. LIMA-to-LAD anastomosis was performed using running 8-0 prolene suture, and paravascular fat was fixed to epicardium with two 6-0 prolene sutures. Coronary probe or flow-probe was not used while or after performing the anastomosis. Before closing chest the LIMA-to-LAD anastomosis and the LIMA itself were examined carefully, and no bleeding or LIMA tension was observed.

**Figure 1:**
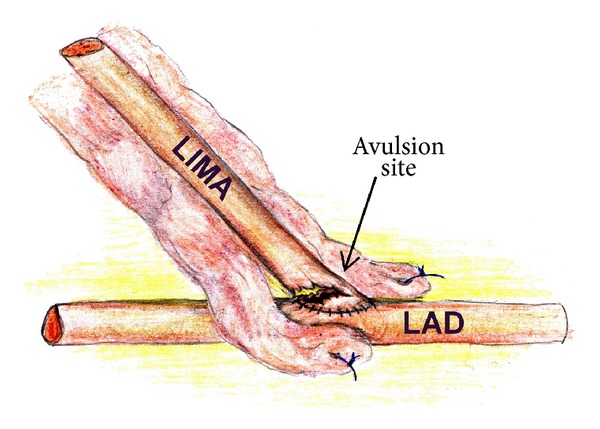
Finding during reexploration. LIMA: left internal mammary artery graft; LAD: left anterior descending artery.

After an uneventful operation the patient was placed to ICU where 1.5 liter of blood drained suddenly through the chest tubes in 1.5 hours after CABG. The patient was returned to the operation room (OR) and reexplored by the same surgeon. Avulsion of the LIMA graft was found at the site of the heel of anastomosis with LAD (Figure 1). There was sufficient length of the LIMA graft, and excessive lung inflation was unlikely to cause tension of the graft. The LIMA itself was examined carefully and had normal external appearance. Technical error was considered to be the cause of LIMA avulsion. The tip of the LIMA graft was resected, and a new LIMA to LAD anastomosis was constructed using CPB and cardioplegia. But in 9 hours of stay in ICU new massive bleeding occurred. The patient was reexplored in the OR again, and similar LIMA graft rupture was revealed. LIMA graft was removed and LAD was bypassed with vein graft using CPB and cardioplegia.

Perioperatively ceftriaxone 2 mg per day was administered intravenously for 2 weeks for syphilis. Early postoperative period was complicated with delirium, deep sternal wound infection, necrotizing pancreatitis, and perforated peptic ulcer. Due to these conditions the patient underwent long mechanical ventilation and tracheostomy, vacuum-assisted closure therapy and secondary sternal wound closure, ultrasound-guided percutaneous catheter drainage for pancreatitis, and surgical repair of the gastric perforation. The patient was discharged home in two months after surgery. Three years after surgery, the condition of the patient is satisfactory.

**Figure 2:**
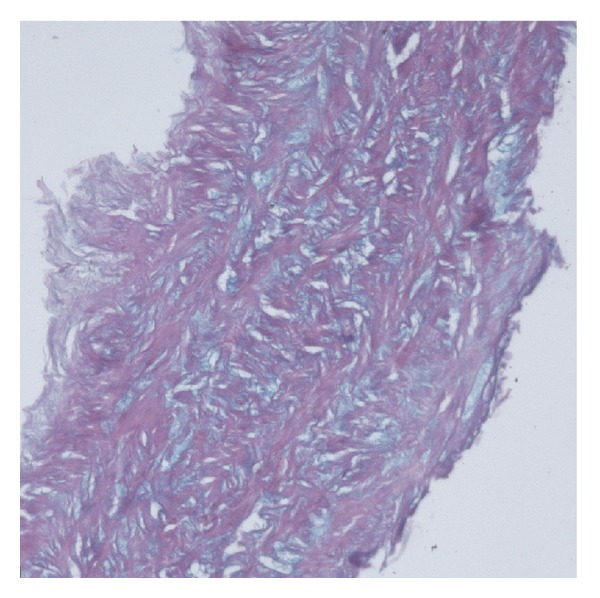
Mucoid degeneration of the LIMA wall. Alcian blue stain (×100).

Histological examination of the resected LIMA showed weakening of the arterial wall possibly due to syphilitic process: mucoid degeneration with mucopolysaccharide accumulation, elastic fibers alteration, and fragmentation (Figure 2).

## 3. Discussion

Syphilis is well known to be the cause of aortitis which tends to result in aortic aneurysms, aortic valve regurgitation, and ostial coronary artery stenosis [1–3]. Proximal saphenous vein graft occlusion after CABG due to syphilitic aortitis was reported [4]. Subclavian, carotid, and innominate arteries also could be affected by the syphilitic process [1, 5, 6], but internal mammary arteries are very seldom involved [3, 7]. Moreover, to our knowledge, it has never been reported to be the cause of LIMA graft avulsion.

Avulsion of a graft is a rare complication of CABG surgery. It can have a number of causes. Several authors reported LIMA graft avulsion after MIDCAB [8–12]. Morritt et al. reported LIMA graft avulsion 4 hours after conventional CABG surgery occurring at the time of weaning from ventilator [13]. Inadequate length of the LIMA, interaction of the artery with the edge of the pericardium, inadequate side branch clipping, and adhesion of the conduit to surrounding anatomical structures such as chest wall, mediastinum, or lung make it susceptible to excessive traction, while cardiopulmonary resuscitation, hyperventilation, coughing, sneezing or weight lifting were named as possible causes in these cases. Mediastinitis was reported to be a cause of saphenous vein graft rupture 2-3 weeks after surgery [14], but potentially it can affect LIMA graft, too. Even without surgery, spontaneous internal mammary rupture has been described in patients with type IV Ehlers-Danlos syndrome [15, 16]. Similar cases were reported to be provoked by catheterization [17].

In our case there were no signs of syphilis prior to surgery such as skin lesion, aortic dilatation, aortic valve insufficiency, or isolated ostial coronary arteries lesion, but blood tests (RPR, EIA, TPPA, and FTA-ABS) were strongly positive for syphilis. LIMA appeared normal during dissection and performing anastomosis with LAD. After first avulsion the LIMA graft was examined carefully and had normal external appearance. Technical error was considered to be the cause of LIMA avulsion. That is why we used the graft again. Nevertheless, further histological examination of the LIMA showed weakening of the arterial wall with such changes as mucoid degeneration with mucopolysaccharide accumulation, elastic fibers alteration, and fragmentation in arterial wall. These findings are not specific for syphilis or any other disease. Similar findings can be found in patients with infectious or systemic arteritis and connective tissue disorders. But there was no systemic manifestation of possible disorder in our 54-year-old patient prior to surgery and during the 3-year follow-up. The only manifestation was LIMA graft rupture. Because of serology strongly positive for syphilis and the absence of another reason of LIMA graft rupture, we suspect syphilis to be the cause of LIMA wall weakening which leads to rupture in our case.

This clinical situation gave us a good practical suggestion: avoid using of a ruptured graft again. Good vein is better than compromised LIMA even if the latest looks externally well!.
